# Cementoin–SLPI fusion protein binds to human monocytes and epithelial cells and shows higher biological activity than SLPI

**DOI:** 10.1038/s41598-018-23680-0

**Published:** 2018-03-28

**Authors:** Paulo C. Maffía, Diego Guerrieri, Ximena Villalonga, Fiorella Caro, Sonia Gómez, Nancy Tateosian, Betiana P. Bogado, Mercedes L. Sánchez, Nella Ambrosi, Eduardo Chuluyan

**Affiliations:** 1 Laboratorio de Microbiología Molecular, Universidad Nacional de Quilmes, Buenos Aires, Argentina; 2 Universidad de Buenos Aires. Facultad de Medicina. Departamento de Microbiología, Parasitología e Inmunología, Buenos Aires, Argentina; 3 Centro de Estudios Farmacológicos y Botánicos-CONICET (CEFYBO), Buenos Aires, Argentina; 4 Departamento de Química Biológica, Facultad de Ciencias Exactas y Naturales, Universidad de Buenos Aires, Buenos Aires, Argentina; 5 Servicio Antimicrobianos, Dpto. Bacteriología, Instituto Nacional de Enfermedades Infecciosas, ANLIS “Dr. Carlos G. Malbrán”, Buenos Aires, Argentina

## Abstract

Secretory Leukocyte Proteinase Inhibitor (SLPI) is an antiinflammatory peptide that blocks the activity of serine proteases, primarily the neutrophil elastase. In an attempt to direct the activity of SLPI on inflamed sites, a chimera consisting of the transglutaminase II substrate domain of trappin 2 (cementoin), and the mature SLPI protein was constructed. Cell attachment and biological activity were compared between SLPI and this chimera. By using whole cell ELISA, fluorescence microscopy and flow cytometry assays we observed that the cementoin-SLPI fusion protein (FP) but not SLPI attached to a human lung epithelial cell line and monocytes. A maximum attachment was achieved 15 min after FP was added to the cell cultures. In an elastase activity assay, we observed that FP retained its antiprotease activity and that at equimolar amount of proteins, FP was more efficient than SLPI in the inhibition. Both, FP and SLPI inhibits IL-2-induced lymphocyte proliferation, however, lower amounts of FP were required to achieve this inhibition. Furthermore, FP binds to mycobacteria and maintained the bactericidal activity observed for SLPI. Overall, these results show that this new chimera is able to attach to the cell surfaces retaining and improving some biological activities described for SLPI.

## Introduction

SLPI and ELAFIN are low molecular weight endogenous serine proteases inhibitors^1^. They are produce by epithelial cells and they are found in mucosal fluids including lung, digestive and genital systems^2–4^. Moreover some myeloid cells may produce them^5^. These serpins can control excessive proteolysis due to the action of neutrophil serine proteases such as elastase, cathepsin G and proteinase-3^6^. Both proteins have been implicated in several physiological and pathological events, such as wound healing, pregnancy, chronic obstructive pulmonary disease, cancer, ischemia reperfusion injury and stroke, among others^7^.

The structure of SLPI and ELAFIN is characterized by the presence of whey acidic protein (WAP) domains^1^. SLPI contains two WAP domains, on the contrary ELAFIN contains only one and it is synthesized from a precursor named Trappin-2. The proteolysis at the C-terminal domain of Trappin-2 generates a WAP domain similar to those found for SLPI. The N-terminal domain of Trappin-2 (38 residues) contains 5 repeated motifs with the consensus sequence GQDPVK identified as a substrate of tissue type transglutaminase-2 (TGase-2). This N-terminal portion is known as the cementoin domain^8,9^ and it is responsible for the covalent attachment of Trappin-2 with various extracellular matrix proteins. It has been reported that Trappin-2, ELAFIN and also SLPI or its chimeras can be covalently linked to extracellular matrix proteins through transglutamination, while retaining their anti-protease capacity^10^. The therapeutic potential use of SLPI has been precluded due to their short half-life in plasma and its inactivation by oxidation or by complexation with neutrophil elastase^11–15^. However, it has been shown that SLPI can inactivate neutrophil elastase when it is bound to elastin^15^, suggesting that binding to membrane or extracellular matrix could protect its structure and perhaps extend its half-life and biological activities. In fact, we have previously reported that a fusion protein (FP), consisting of the N-terminal domain of Trappin-2 (cementoin) and mature SLPI, protected the corneal from a noxa, preventing the development of a serious corneal abscess in rats^16^. However, this effect was not observed when SLPI was administered to the rats. Therefore, we have speculated that the addition of cementoin to the SLPI structure favors the attachment of SLPI to cell surfaces and transforms the serpine into a new peptide with different properties. In the present study we analyzed and compare the binding ability of the new FP, that comprises of cementoin peptide fused to mature SLPI. We showed by different techniques that this FP, but not SLPI, was able to attach to the cellular surface of the human lung cell line A549 and monocytes. Furthermore, we showed that FP retained and increased some biological activities described for SLPI.

## Results

### Binding of FP to A549 cell surface

**Figure 1:**
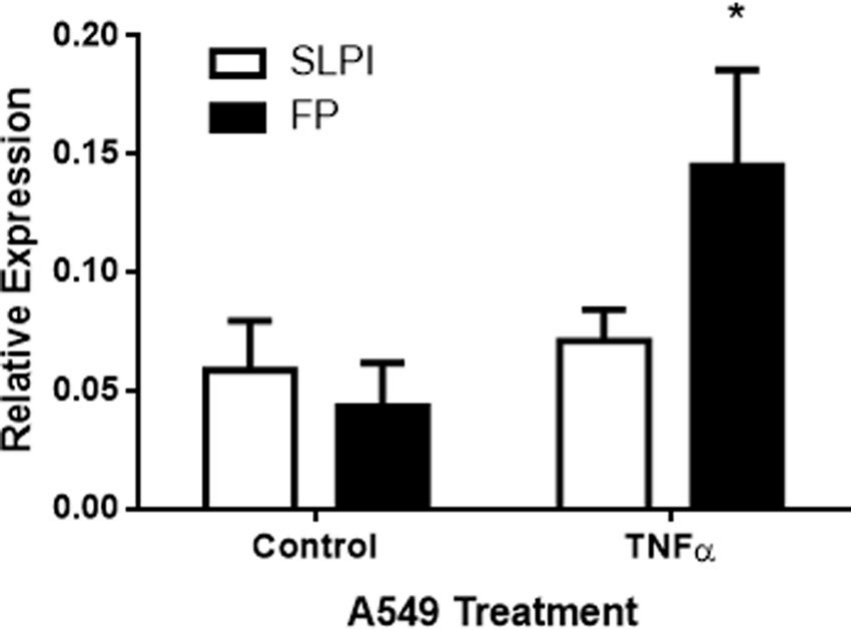
Adhesion of SLPI and FP to the surface of A549 cells. Cells were untreated (control cells) or pre-treated with TNF-α (10 ng/ml, 24 h). Then, cell monolayers were incubated with SLPI or FP (4 μg/ml, 37 °C, 1 hour). Cells were washed and labeled with a mouse monoclonal antibody against histidine, followed by a secondary anti-mouse peroxidase-labeled. Quantification was performed by reading the OD at 452 nm in an ELISA reader. Data represent mean ± SEM of the relative expression (OD of cells with SLPI or FP minus OD of untreated cells). *p < 0.05 vs untreated control cells. Student *t* test for unpaired data.

**Figure 2:**
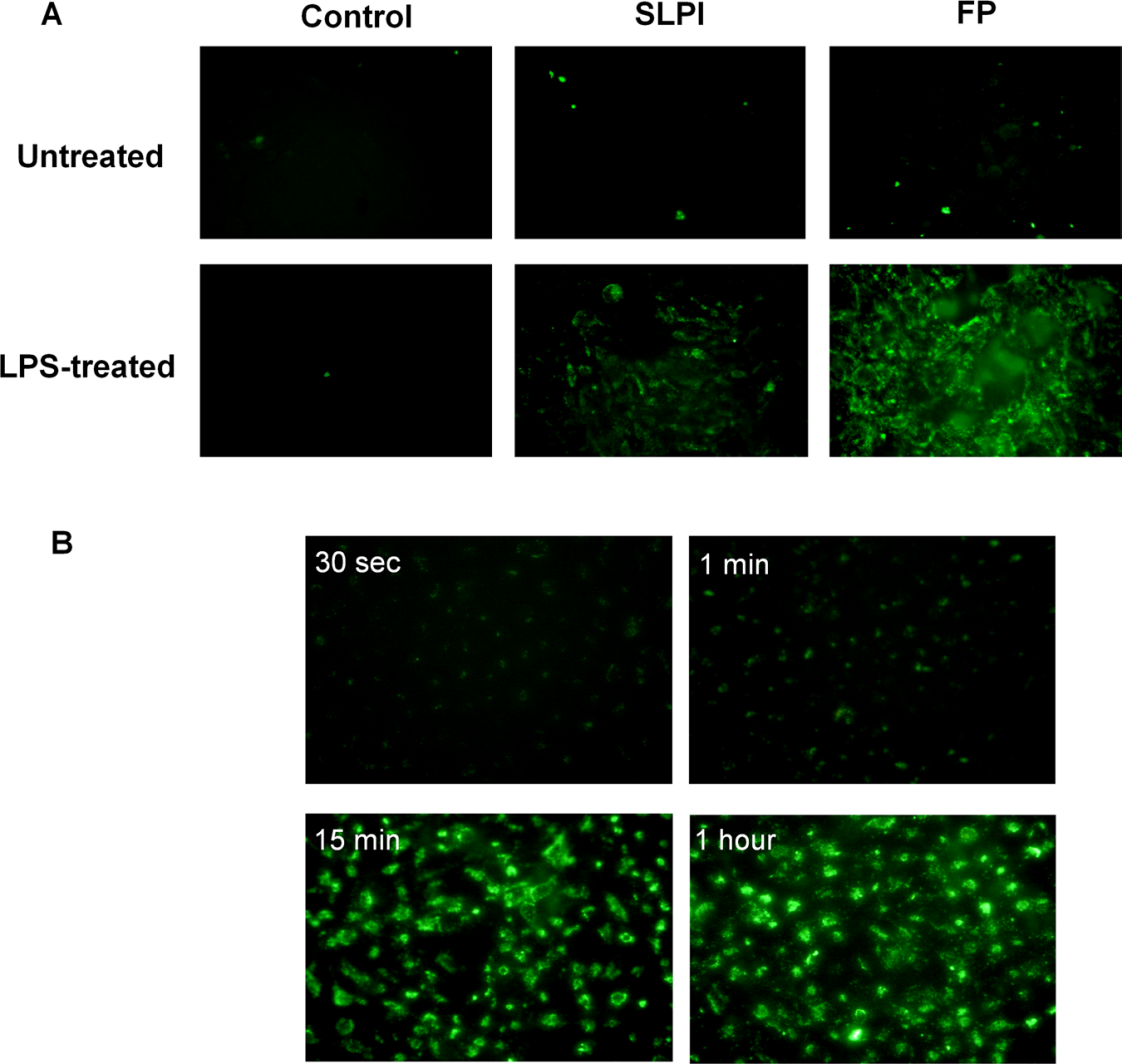
Fluorescence microscopy image of A549 cells incubated with SLPI or FP. (**A**) A549 cells were pretreated with 400 ng/ml of LPS (24 h, 37 °C) and then incubated with of SLPI or FP (4 μg/ml, 37 °C, 1 hour). Cells were washed and labeled with an anti-histidine antibody conjugated with FITC. A representative fluorescence microscopy image from 3 different independent experiments is depicted. (**B**) Time kinetics binding of FP to A549 cell line. LPS-treated A549 cells were incubated with FP (1.3 μg/ml, 37 °C) at different time points. After 0.5, 1, 15 and 60 minutes, cells were washed and then sequentially incubated with a mouse antibody against cementoin and then with an anti-mouse monoclonal antibody conjugated with FITC. A representative fluorescence microscopy image from 2 different experiments is depicted.

**Figure 3:**
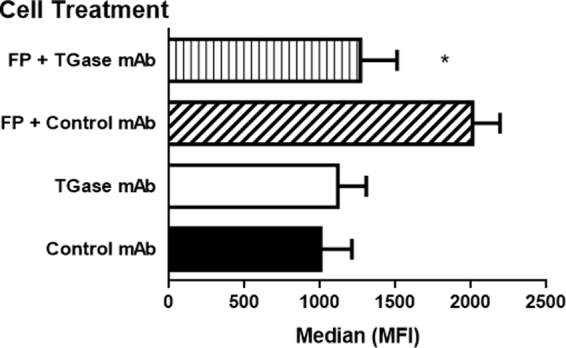
FP crosslinking to cell membrane TGase-2. A549 cells were treated with LPS (24 h, 37 °C). Cells were detached and pre-incubated 30 min at RT with mAb to tissue TGase2 or irrelevant isotype control mAb. Following, cells were treated or not with FP (4 μg/ml, 37 °C, 15 min). Afterwards, cells were stained with a mAb against anti-6 His tag and fluorescense were measured with flow cytometer. Data represent the mean ± SEM of MFI of 3 independent experiments. *p < 0.05 paired *t* test compared to FP + control mAb.

It has been reported that human epithelial alveolar cell line A549 expresses tissue TGase-2^17^, which is increased by LPS and TNF-α^18–20^. In this context we first compared the binding ability of SLPI and FP to this cell line. Untreated or TNF-α-treated A549 cells were *in vitro* cultured with FP or SLPI and their binding to the cell surface was examined in a whole cell ELISA assay. Figure 1 shows that binding of SLPI or FP to untreated cells is low and similar. However, TNFα-treated A549 cells increased the bind of FP but not SLPI to the cell surface (Fig. 1). In order to confirm that pro-inflammatory stimulus favors the binding of FP but not SLPI on A549 cells, we performed an immunofluorescence experiment on LPS-treated A549 cells (Fig. 2). Like the whole cell ELISA assays, we did not observed binding of FP or SLPI to the surface of untreated cells. Clear fluorescent labelled cells were observed when cells were pre-treated with LPS and incubated with FP (Fig. 2A). However, the fluorescent label was much lower when LPS-treated cells were incubated with SLPI (Fig. 2A). Following, we performed a time kinetic assay of the FP attachment to the cells membrane. For this purpose, FP was incubated during different time lapses with LPS-pretreated A549 cells. Figure 2B shows that cell fluorescence was not observed at 30 seconds, while it was observed slightly dim at 1 min and intense at 15 and 60 minutes. Since there were no differences between 15 and 30 min of incubation, we assumed that 15 min was the time necessary to saturate the attachment of the protein to the surface of the cells. Taking into account previous bibliography, we hypothesized that an anti-TGase mAb could block the FP binding site. Therefore, in order to determine whether cell membrane TGase-2 crosslinks FP, we performed a binding experiment of FP to A549 in the presence of an anti-tissue TGase-2 mAb. For these experiments, LPS- treated A549 cells were preincubated for 30 min with an anti-TGase-2 mAb. Afterwards, FP was added, incubated for 15 min and binding was detected with an anti-His-6XTag PerCP labelled mAb by flow cytometry as described in Materials and Methods. Figure 3 shows that cells incubated with FP displayed lower mean fluorescence intensity when they were pre-incubated with an anti-TGase-2 mAb compared to FP treated cells pre-incubated with an irrelevant isotype control mAb. This result suggests that cell membrane TGase crosslinks FP.

### Binding of FP to leukocytes cell surface

In order to evaluate the capacity of FP to bind cell surfaces in the context of inflammation, the attachment of FP to human peripheral blood mononuclear cells (PBMC) was analyzed.

**Figure 4:**
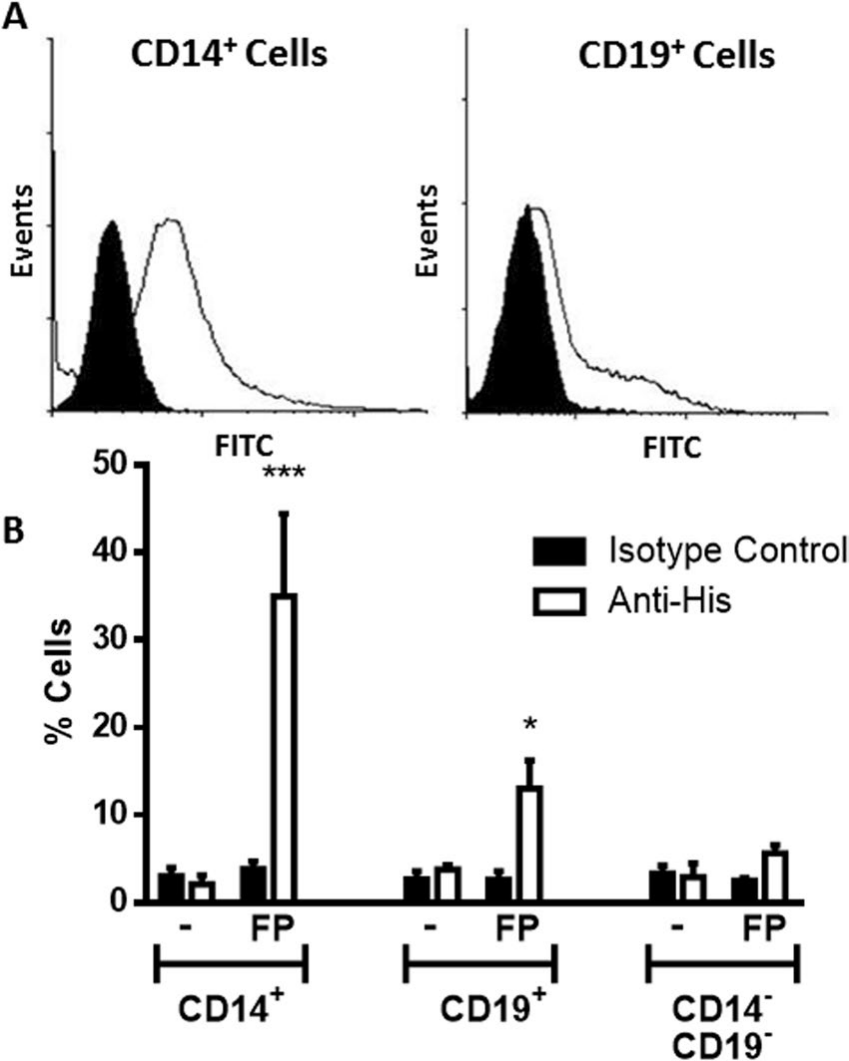
Binding of FP to human mononuclear cells. PBMCs from healthy volunteers were incubated with FP (4 μg/ml, 1 h, 4 °C). Then cells were washed and incubated (1 h, 4 °C) with a mouse IgG1 anti-histidine mAb (clear histogram) or isotype control antibody (black histogram). Following, cells were washed and stained with a rabbit FITC-IgG1 anti-mouse polyclonal antibody (SC Biotechnology, USA). Finally, cells were stained with a mouse PE anti-human CD14 and APC anti-human CD20 mAbs. Cells were then analyzed by flow cytometry. A representative experiment out of three independent experiments is shown in A and the mean ± SEM is shown in B. *p < 0.05; ***p < 0.001 Two way ANOVA, uncorrected Fisher’s LSD test.

Human PBMC were isolated and incubated with FP for one hour. Afterwards, cells were stained with CD14 or CD19 mAb and FITC anti-histidine antibody in order to detect the binding of the FP on human monocytes (CD14^+^), B lymphocytes (CD19^+^) and T cells (CD14^−^ and CD19^−^). The cell flow cytometry analysis showed strong staining on CD14^+^ cells and mild on CD19^+^, while no binding on CD14^−^ and CD19^−^ cells (Fig. 4A and B).

### Biological Activity

**Figure 5:**
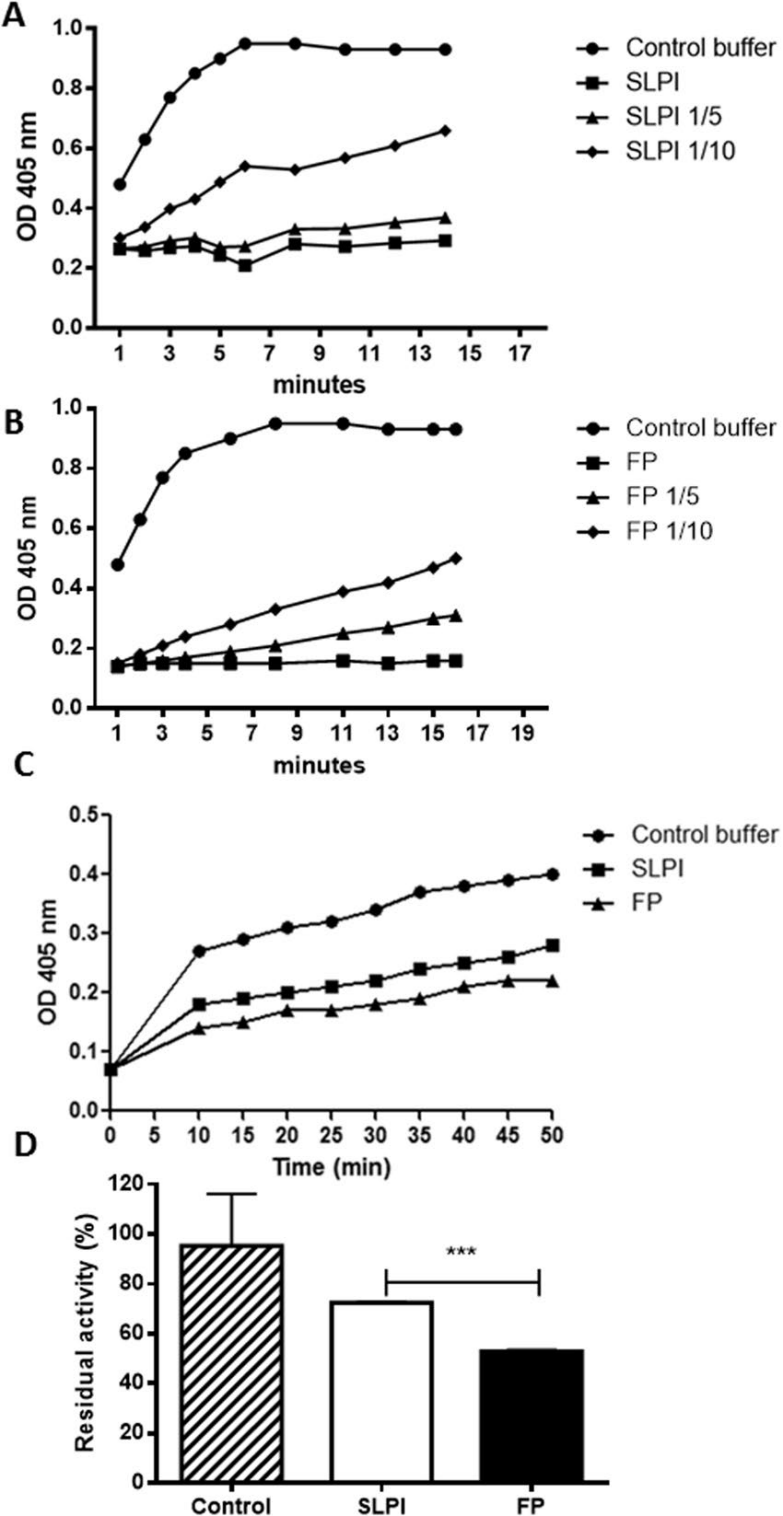
Quantification of SLPI and FP elastase inhibitory activity. Time kinetic curve for the inhibition of elastase proteolytic activity by SLPI (**A**) and FP (**B**). The activity was analyzed by quantifiying the amount of p-nitroanilide released after cleavage of an elastase specific substrate. The OD was measured at 405 nm every 2 min. (**C**) Time kinetic curve for the inhibition of elastase proteolytic activity by equimolar concentrations of SLPI and FP. The OD was measured at 405 nm every 5 min for 50 min. (**D**) Residual proteolytic activity of elastase alone or co-incubated with SLPI or FP. The residual activity was calculated as the ratio of rate of substrate hydrolysis in the presence of inhibitor to the rate of substrate hydrolysis without inhibitor (control). Data represents the mean ± SD for three experiments. Unpair *t* test p < 0.0001.

Finally, we compare the biological activity of FP and SLPI by analyzing the antiprotease activity and their ability to inhibit lymphocyte cell proliferation in an equimolar and comparable assay as described in Materials and Methods. Figure 5A and B shows that SLPI and FP have anti-protease activity since both proteins reduced the cleavage of the elastase-specific substrate. The inhibition activity is decreased gradually by reducing the concentration of the proteins. However, at equimolar amounts of protein, the FP displayed higher activity than SLPI (Fig. 5C and D).

**Figure 6:**
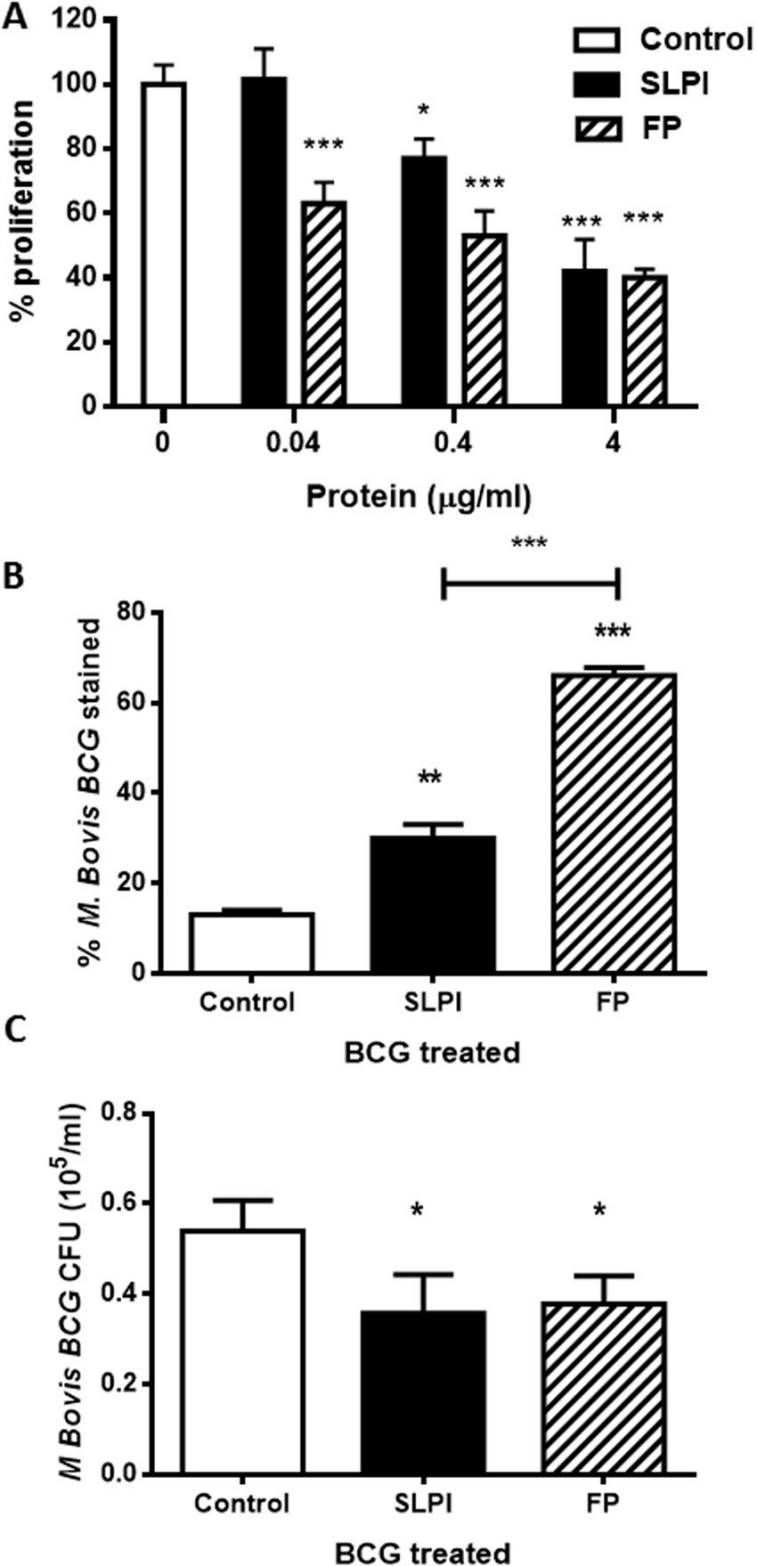
Biological activity of FP. (**A**) Human PBMC proliferation. PBMC (10^5^ cells/well) were cultured for 5 days in the presence of IL-2 (8 ng/ml). Cells were untreated or treated with different concentrations of SLPI or FP. Proliferation was measured by [3 H]TdR incorporation during the final 18 h of culture. Data are expressed as mean ± SEM percentage proliferation relative to the controls. Controls were PBMCs culture with IL-2 (98 ± 3.5%; n = 7). *p < 0.05; **p < 0.01 and ***p < 0.001 ANOVA pos hoc Dunnett test for multiple comparisons. (**B**) Binding of SLPI and FP on *M. bovis BCG*. For binding experiments, Bacterial particles (10^5^) were incubated with control buffer, SLPI (4 μg/ml) or FP (4 μg/ml) for 2 hours at 37 °C. After incubation, the cells were washed and incubated with Penta-His Alexa Fluor 647 conjugate (30 min, 4 °C) to detect histidine-tagged SLPI or FP. The cells were fixed and binding was detected by flow cytometry. The data represent the percentage of mean ± SEM of three experiments. **p < 0.01, ***p < 0.001 Sidak’s multiple comparisons test. (**C**) Microbicidal activity against *M. Bovis BCG*. 50 μl volumes of mid–log phase bacteria at 1 mg/ml in Sauton buffer was added to 96-well polystyrene microplates in triplicate, together with 4 μg/ml of SLPI, FP or control buffer. After overnight incubation at 37 °C, the number of colony-forming units was determined by plating serial dilutions onto 7H11 agar plates. Data represent the mean ± SEM of the percentage of microbicidal activity of two separate experiments. *p < 0.05, Tukey’s multiple comparisons test.

In a previous work we showed that SLPI inhibits lymphocyte proliferation^21^. This effect depends on the presence of monocytes^21^. Therefore, based on the binding of FP mainly on CD14^+^ cells (Fig. 4), we compared the activity of both peptides on lymphocyte proliferation. Human PBMC were isolated and incubated in the presence of different concentrations of FP or SLPI and proliferation was induced by IL-2 (8 ng/ml). Figure 6A shows that 4 and 0.4 μg/ml, but not 0.04 μg/ml of SLPI reduced cell proliferation. However, for FP the inhibition on cell proliferation was observed even at 0.04 μg/ml. Another relevant function of SLPI is its bactericidal activity. Indeed, we have previously shown that SLPI binds and has bactericidal effect against *Mycobacterium Bovis* BCG^22^. When FP was incubated with BCG, we observed that FP not only retained the SLPI binding capacity to BCG but also increased it (Fig. 6B). However, when microbicidal activity was tested, we observed that both proteins showed a similar anti-mycobacterial effect (Fig. 6C).

## Discussion

The biological function of the transglutaminase-catalyzed covalent linking of trappin-2/ELAFIN to extracellular cellular matrix (ECM) proteins is to protect them from degradation by neutrophil serine proteases, so helping maintain tissue integrity during inflammation and/or tissue remodeling^8,23^. The posibility of SLPI to avoid serine proteases degradation seems to be more limited than other serpins with tranglutaminase binding motifs, such as Trappin-2. The new FP generated by our group is thought to give SLPI the possibility to be more resistant to the proteolysis, as cementoin was added to the SLPI structure. In fact, since a mAb against TGase was able to partially block the binding of the FP to the cell surface, it is highly probable that cell surface TGase-2 targets and crosslinks FP to cell surface membrane. Thus, FP retains and improves some biological activities described for SLPI such as elastase inhibition and the inhibitory effect on lymphocyte proliferation.

One of the problems with the SLPI-based therapies is probably the relative short half-life of this protein, as it degrades shortly after being injected or applied in different ways^11,24^. Furthermore, SLPI can be internalized and distributed throughout the cytoplasm and nucleus of monocytes, acquiring anti-inflammatory properties but reducing its concentration at the inflammatory microenvironment^25^.

In the present work we can see a slight binding of SLPI to LPS-treated A549 (Fig. 2). This was not unexpected since it has been demonstrated that SLPI is also a TGase substrate, and that it can be crosslinked to ECM proteins while retaining its elastase inhibitory activity^10^. However, this binding of SLPI to LPS-treated cells, was not as strong as FP. In fact, Baranger and co-workers also showed that a fusion protein consisting of cementoin fused to the domain 2 of SLPI was a far better TGase substrate than is SLPI alone^10^ and also retains its ability to inhibit neutrophil serine protease cathepsin G. In agreement with these findings, our protein consisting of the cementoin peptide fused to the complete mature SLPI protein, showed better neutrophil elastase inhibitory activity than SLPI alone.

One of the main findings of our study, it is the demonstration that binding was higher when the epithelial cell line was treated under pro-inflammatory conditions, such as TNF-α and LPS. This could be explained since different cytokines, growth factors and hypoxic stimulus up-regulates tissue TGase-2 expression^26^.

When the cell surface attachment of FP to human immune cells was studied, a protein binding to monocytes and B cells was observed (Fig. 4). Though the expression and function of TGase-2 on monocytes have been better characterized, to our knowledge this issue has not been thoroughly addressed on B cells^27,28^. Dendritic cells, which like B cells are professional antigen processing cells, express tissue TGase-2^29^. Therefore, it is not unlikely that B cells, such as monocytes and dendritic cells also express TGase2. This issue and its function on B cells deserve further investigation but it is out of the scope of the present work.

*A priori*, we could speculate that FP and Trappin-2 would display different activities, based on the fact that the latter has only one WAP domain and it has been described to exert a more restricted anti-protease activity^30^. Although, this comparison between FP and Trappin-2 could arose interest and shed light on the understanding of the role of cementoin, it is not the aim of the present study. Further experiments are needed to address this specific issue.

FP displayed better enzymatic and biological function than SLPI when compared. The reasons for these differences could be due to: i) the binding of cementoin to TGase that triggers some biological events associated with the signaling through TGase-2; ii) a more stable structure of FP than SLPI. Indeed, it is known the importance of the structures and the conformational changes of serpins for the correct function of them^31^. In any case, the covalent anchor of SLPI to the site of inflammation, through the cementoin peptide, could be a useful tool for a SLPI based therapy, because in this way it could increases its local concentration and displays a targeted activity. In fact, in a previous *in vivo* work in a model of corneal inflammation, we observed that FP but not SLPI resolved the inflammatory process^16^.

The biological activities of SLPI and Elafin are not circumvent to their anti-proteases and anti-inflammatory activities. Other well characterized function of SLPI is its wide spectrum microbicidal activity, wound healing and antitumor effects. Although, we have not yet assessed the activity of FP in wound healing and antitumor activity, we observed that FP displayed a bactericidal activity against mycobacteria as good as SLPI^22^. However, the binding ability of FP to mycobacteria was twice higher than SLPI, which has been described as a secreted pattern recognition receptor for mycobacteria^22^. This difference is relevant since it is probable that the clearance of mycobacteria opsonized with FP could be more efficient than SLPI.

In light of these findings this FP could arise as a new therapeutic tool, but further studies are needed to know its therapeutic potential in *in vivo* models.

## Materials and Methods

### FP gene construction

As it was previously described for SLPI^32^ cementoin mRNA was extracted from HeLa cells (epitheloid cervix carcinoma) and reverse-transcribed to cDNA using oligo-dT primers with MMLV-RT (Promega, Madison, WI) according to specifications of the manufacturer. Two pairs of modified PCR primers (forward primer GTTCTACATATGGCTGTCACGGGAGTT and reverse primer TTAAAGGTCAAGATAAAGTCAAAAAGCTT) were used to generate the complete open reading frame of the cementoin peptide from the obtained cDNA. The SLPI and cementoin cloned genes were amplified by PCR with modified primers, these primers created new recognition sites for restriction enzyme and an ATG (Met) translation initiation codon on the 5′ end. The plasmids, pGEMT-SLPI and PGEMT-cementoin were digested with the restriction enzimes *ApaI* and *HindIII* (Promega, Madison, WI) and cementoin and SLPI fragments were incubated together in equimolar amounts in a ligation reaction with the enzyme T4 DNA ligase (Promega). This insert was then ligated to the pET22b^+^expression vector (Stratagen).

The pET-Cementoin-SLPI vector was purified and electroporated in the *E. coli* expression strain Origami (Novagen, Inc., an Affiliate of Merck KGaA, Darmstadt, Germany). Origami host strains have mutations in both the thioredoxin reductase (trxB) and glutathione reductase (gor) genes, which greatly enhance disulfide bond formation in the cytoplasm.

### Purification of the recombinant proteins

SLPI and FP were expressed and purified as described previously^16^. Briefly, Bacterial culture was grown by shaking at 37 °C until an optical density of 0.6 measured at 600 nm. The expression of the recombinant protein was induced by adding IPTG (isopropyl thiogalactoside, 1 mM) for 3 hours of induction at 28 °C with continuous stirring. Finally, the culture was centrifuged (7000 rpm for 7 min) and the pellet was split and stored at −20 °C.

Bacterial pellets were resuspended in 2 ml lysis buffer (50 mM NaH_2_PO_4_, 1 M NaCl, 10 mM Imidazole, pH = 8) and then sonicated. The lysate was centrifuged (10000 g, 30 min, 4 °C), the soluble fraction was recovered and added to 1 ml of Nickel-nitrilotriacetic acid (Ni-NTA) agarose beads (Qiagen GmbH, Hilden, Westphalia, Germany). Subsequent washes with increasing concentration of imidazole (20–40 mM) were performed and the protein was eluted with 250 mM imidazole, pH = 8. Finally, the eluted samples were dialyzed in phosphate buffer overnight at 4 °C. For LPS removal, polymixin-agarose was used. Possible residual LPS was confirmed by performing a Limulus test assay.

### Quantification of protein concentration

Protein quantification was performed using the MicroBCA (Pierce, USA) kit following the instructions of the manufacturer. The assay was performed in an ELISA plate, which was subsequently read on a reader plate at 550 nm.

### Acrylamide gel electrophoresis

Aliquots from the different purification steps were heat denatured (5 minutes at 95 °C) in buffer Drill (SDS 0.5% w/v glycerol 2.5% v/v, 2-β-mercaptoethanol, 1.25% v/v). Then, the different samples were loaded in a polyacrylamide gel (18%). The run was performed under denaturing conditions at 120 V for 1 h. The gel was stained with Coommassie-Blue and destained according to standard techniques. Finally, the gel photograph was digitized for further analysis.

### Measurement of elastase activity

The proteolytic activity of elastase was quantified as the absorbance produced by the cleavage of the chromogenic substrate N-methoxysuccinyl-Ala-Ala-Pro-Val p-nitroanilide (Sigma, MA). The inhibition assay was performed in an ELISA plate. For the quantification of the elastase inhibitory activity of each protein, 0.5 µl of elastase (4 pmol/μl) was incubated with FP or SLPI (0.2 pmol), for 15 min at 37 °C. Then, 0.6 mM of the chromogenic substrate diluted in Tris buffer (50 mM) at pH = 7.5 was added. The product of the reaction, p-nitroanilide was measured at 405 nm every 10 min in the ELISA reader (Multiskan Labsystem, San Diego, CA). The amount of substrate cleaved was obtained using the Lambert-Beer law.

### Cell line

The human epithelial cell line generated from alveolar lung adenocarcinoma A549, and was grown in HAM F12 medium (GIBCO BRL, USA), 10% inactivated fetal bovine serum (GIBCO BRL), glutamine (2 mM) and gentamicin (40 µg/ml), at 37 °C and 5% CO_2_. All tests were conducted under conditions of sub-confluence (80–90%), with an initial number of 5 × 10^5^ cells/ml culture flask (25 cm^2^), unless otherwise indicated. This cell line expresses transglutaminase II^33^ and LPS or TNF-α induce an increase in its expression^19,20^. Therefore, for some experiments, cells were treated with LPS (24 h, 400 ng/ml) (Sigma, MA) or TNFα (24 h, 40 ng/ml).

### Immunofluorescence assay

The adhesion of the FP to the surface of A549 was analyzed by fluorescence microscopy. A549 cells (3 × 10^4^) per well were seeded in 400 µl of HAM F12 medium (GIBCO BRL), 10% inactivated fetal bovine serum (GIBCO BRL), glutamine (2 mM) and gentamicin (40 µg/ml) in a 48 well plate. Some of the wells were incubated for 24 h with LPS (400 ng/ml) (Sigma), at 37 °C and 5% CO_2_. This LPS was previously activated by 2 cycles of 56 °C 10 min and 10 min more at 4 °C. After the incubation time, the medium with LPS was withdrawn and the cells washed. Media containing FP, SLPI 4 µg/ml or the equivalent volume of dialysis buffer (as a control) was added to the monolayers for 15 seconds to 1 hour, depending on the experiment at 37 °C. Afterwards the monolayers were washed and incubated with fetal bovine serum to a final concentration of 2%, for 10 min (blocking), washed and finally incubated with a primary antibody against the histidine tag (His-probe (H3), Santa Cruz Biotechnology Inc, Dallas, TX) for 40 minutes at room temperature. Then, the medium was removed, washed, and secondary antibody (FITC-conjugated goat anti-mouse, Caltag, Buckingham, UK) was added for 40 minutes in the dark at room temperature. Finally, the monolayers were washed and the fluorescence was observed under the fluorescence microscope (Nikon TE200). For some experiments, FP crosslinking to TGase on A549 cell surface was assessed by Flow Cytometry (Accuri 6 C plus, BD Biosciences, San Diego, CA) in the presence or absence of a mouse mAb to human TGase2 (4G3, Santa Cruz Biotechnology, Inc). For these experiments, LPS-treated cells were detached and incubated with the mAb to TGase or irrelevant isotype control mouse mAb (B-6, Santa Cruz Biotechnology) for 20 min at RT. Following, cells were washed and FP (4 μg/ml) was added during 15 min at 37 °C. Then, cells were washed and anti-6X His tag antibody (PerCP, ab117496, Abcam) or isotype control mouse IgG1 (PerCP, ab118658) was incubated for 45 min, washed and fluorescence read it by Flow Cytometer.

### Whole live cell ELISA

A monolayer of semi-confluent A549 cells were treated or not with TNF-α (500 unit/well, 24 hour) in a 96-well flat bottom plate. Then, the medium was removed, cells washed and FP or SLPI (4μg/ml) were added and incubated at 37 °C for 1 hour. Afterwards, the cells were washed and the anti-SLPI (HBT) antibody (0.8 mg/ml, 90 minutes, 37 °C) was added. The plate was then washed and incubated with anti-mouse peroxidase-labeled antibody (Biorad Laboratories Inc., Hercules, CA) for 1 hour at 37 °C. Finally, the plates were washed with TMB solution. The reaction was stopped with 50 µl sulfuric acid and read at 450 nm in an ELISA reader (Multiskan Lab System).

### FP treatment of human immune cells

For these experiments, human peripheral blood mononuclear cells (PBMCs) were isolated by Ficoll gradient and monocytes were further isolated by CD14 dynabeads. Monocytes were stimulated with LPS (100 ng/ml) for 3 hours at 37 °C. Cells were then centrifuged at 1000 rpm for 10 minutes and washed 2 times with 0.5% FCS in PBS. The cells (5 × 10^5^ cells/ml) were then incubated with 2 μg/ml of FP in RPMI medium at 4 °C for 60 minutes, after which another 2 washes were performed.

### Flow cytometry analysis

Cells were treated or not with FP, afterwards nonspecific binding was blocked using 2% FBS RPMI medium and incubated for 10 minutes at 37 °C. IgG1 anti-histidine mouse monoclonal antibody (Santa Cruz Biotechnology) was then incubated with a final concentration of 3 μg/ml for 1 hour at 4 °C. Cells were then centrifuged, washed and incubated with the FITC-conjugated IgG1 rabbit anti-mouse polyclonal secondary antibody (0.5 μl/1 × 10^6^ cells, Santa Cruz Biotechnology) in 2% inactivated FBS-PBS medium for 1 hour at 4 °C in the darkness. Finally, the anti-human mouse monoclonal PE anti-CD14 and APC anti-CD19 were used to identify monocytes and B lymphocytes respectively (following recommendations of the manufacturer). Cell populations were incubated with the secondary antibody alone and analyzed to rule out false positives. Finally, fluorescence intensity was analyzed using a FACStar Plus (BectonDickinson, BD Biosciences, San Diego, CA) and dead cells were excluded by gating with propidium iodide.

### Cell proliferation assay

Peripheral blood mononuclear cells (PBMC) were cultured in RPMI-1640 10% FBS at 37 °C for 5 days with IL-2 (8 ng/ml) and treated with SLPI or FP (0.04, 0.4 and 4 µg/ml) during which they were pulsed with [3 H] thymidine (1 μCi/well, specific activity 5 mCi/mmol; PerkinElmer, Life Sciences, Boston, MA) for the final 18 hours. Cells were harvested using a multi-well cell harvesters and thymidine incorporation was measured with a beta-counter.

### Binding and antibacterial activity to BCG

Binding of SLPI or FP to M. bovis BCG and the anti-mycobactericidal activity were performed as described previously^22^. In brief, suspensions were incubated with SLPI or FP (2 h, 37 °C) and then incubated with Penta-His Alexa Fluor 647 conjugate (30 min, 4 °C) to detect rhSLPI histidine-tagged protein. Cells were washed, fixed (1.5% paraformaldehyde) and binding was detected by flow cytometry. For the antibacterial activity, bacterial suspensions (1 mg/ml) were treated (16 h, 37 °C) with rhSLPI, FP or control buffer. Samples were taken at 24 hours for colony forming unit detection and viable bacteria were detected by plating onto 7H11 agar plates after 2–4 weeks.

### Statistical analysis

All statistics were analyzed in GraphPad Prism. Unpaired *t*-tests and pair *t* test were used to compare means as stated in each Figure legend. Two way ANOVA, uncorrected Fisher’s LSD test was used for binding of proteins to human mononuclear cells. ANOVA pos hoc Dunnett test for multiple comparisons and Tukey’s multiple comparisons test was used for evaluating biological activity of FP. A p-value < 0.05 was considered significant. Graphs were generated by GraphPad Prism (GraphPad, Inc., La Jolla, CA).

### Ethics Statement

Human peripheral blood mononuclear cells were isolated from healthy volunteers. All participants provided a written informed consent for the collection of samples and subsequent analysis and the protocols conducted in this work were approved by the Ethical Committee of the Hospital Muñiz and the International Review Board Fundación Huésped. All experiments were performed in accordance with relevant guidelines and regulations.
